# Determinants of Patient Use of Telemental Health Services: Representative Cross-Sectional Survey From Germany

**DOI:** 10.2196/70925

**Published:** 2025-06-13

**Authors:** Ariana Neumann, Hans-Helmut König, André Hajek

**Affiliations:** 1 Department of Health Economics and Health Services Research, University Medical Center Hamburg-Eppendorf, Martinistraße 52, Hamburg, 20246, Germany, 49 40741054202, 49 4074104026

**Keywords:** telemental health, telepsychiatry, teletherapy, telemedicine, digital health, patient use, service use

## Abstract

**Background:**

Telemental health services effectively address major challenges in mental health care delivery. To maximize the potential of the services, it is essential to facilitate patient use and reduce use disparities. Nevertheless, determinants of patient use of telemental health services have been scarcely investigated thus far.

**Objective:**

We aimed to identify determinants of patient use of telemental health services since the onset of the COVID-19 pandemic and in the last 4 weeks.

**Methods:**

In December 2023, we conducted a cross-sectional, quota-based (gender and age group) online survey. The sample comprised individuals aged 18 to 74 years, who had been using mental health services since March 2020 (n=2082). Telemental health service use was assessed using items that inquired whether individuals had used the services since March 2020 or currently (in the last 4 weeks). Logistic regressions were computed to test the associations of socioeconomic, access, health, COVID-19–related, psychosocial, and service factors, as well as personality and provider characteristics with patient use.

**Results:**

Younger age, a more positive patient attitude toward telemental health services, a more positive provider attitude toward using the services, and higher provider skills for using the services were positively associated with patient use of telemental health services since the onset of the COVID-19 pandemic. When exclusively looking at current use, positive associations with full-time employment, lower neuroticism, a more positive provider attitude toward the services, and use of the services to avoid stigmatization, long waiting times, or inconvenient scheduling were observed. Access, health, and COVID-19–related factors were not associated with patient use (since the onset of the COVID-19 pandemic and currently).

**Conclusions:**

Beyond socioeconomic factors, personality, and a positive patient attitude toward the services, patient use of telemental health services was associated with a positive provider attitude toward using the services and higher provider skills for using the services, which underscores the need for provider support and training in telemental health care. Furthermore, avoiding stigmatization and higher convenience of the services were associated with patient use, which highlights the substantial potential of the services to address current mental health care challenges.

## Introduction

### Background

Numerous reviews reported unmet needs and critical gaps in mental health care provision worldwide [1-3]. Critical factors contributing to this issue include provider shortages, ineffective collaboration, organizational issues, limited access to care, mental health stigma, and discrimination [4-6]. Inadequate mental health treatment can lead to several adverse outcomes, such as chronic and severe disease courses, greater risk of suicide or premature mortality, and high economic costs [2]. Considering that more than a billion people are affected by mental or addictive disorders globally [7], it is crucial to improve and transform the future landscape of the mental health care provision.

Telemental health services offer a potential solution to major challenges in the provision of mental health care [8,9]. Telemental health may be defined as the use of telecommunications or videoconferencing technology to provide mental health services [10]. The delivery of telemental health services can be carried out via synchronous services (eg, video or telephone calls), asynchronous services (eg, mobile apps, web-based interventions, and email), or hybrid combinations [11]. The services may enhance access to mental health care, decrease waiting times, save costs, reduce stigmatization, and help to facilitate screening, diagnosis, and monitoring of mental illnesses [8,9,12]. Furthermore, multiple past studies confirmed the effectiveness of synchronous as well as asynchronous telemental health services and found comparable results to in-person services in terms of clinical effectiveness, diagnostic reliability, efficacy, working alliance, attrition rates, and patient satisfaction [13-18].

The strengths of telemental health services played a significant role during the COVID-19 pandemic, when in-person mental health services were restricted. Telemental health services allowed the continuation of mental health care while minimizing physical contact and the risk of infection. Consequently, telemental health service use increased remarkably worldwide [19,20]. For instance, 90.5% of mental health professionals from 100 countries (n=1206) stated to have either started or increased telemental health service use since the onset of the COVID-19 pandemic. Also in Germany, which is the focus of our study, the COVID-19 pandemic accelerated the digital transformation of the health care system, supported by the adoption of laws such as the Digital Healthcare Act [21] and the Digital Health Applications Ordinance [22]. The proportion of contract physicians and psychotherapists using telemedicine in Germany increased from 6.1% in 2019 to 24.6% in 2021, with telemental health services accounting for up to 86% of this increase [23].

Similar to other high-income countries, the German mental health care system struggles with challenges such as provider shortages alongside increasing need for care, regional differences in supply density (eg, underserved rural areas), long waiting times for treatment, stigmatization and discrimination, ineffective collaboration, and the provision of (long-term) care for severe cases [24,25]. Telemental health services have the potential to help overcome these obstacles. For example, the services can deliver care regardless of location, are accessible to mobility-restricted patients, reduce travel and waiting times, and provide greater levels of anonymity (eg, [8,12]). Therefore, increasing the use of telemental health services may be crucial for improving the quality, efficiency, and accessibility of mental health care in the future.

Postpandemic research highlights the consistency of international telemedicine use and its potential to address mental health care challenges beyond those presented by the COVID-19 pandemic [26,27]. Despite the continued use of telemedicine, disparities in use and acceptance of the services persist. Differences in access, socioeconomic and health factors, technological difficulties, the digital divide, privacy and security concerns, regulatory and legal issues, and clinical limitations were identified as potential causes of these disparities [26,27]. Furthermore, higher attrition rates for telemental health services were observed for certain remote service delivery models and among providers with lower levels of experience [18]. Reducing variation in use and ensuring consistent adherence to telemental health services is crucial for the effectiveness of future services, which serve as a meaningful tool in addressing current mental health care challenges.

Identifying determinants of patient use of telemental health services may help to minimize use disparities and prevent attrition. Nevertheless, existing research regarding this topic is limited, particularly from Germany. In a recently published systematic review, we included 10 studies on determinants of patient use of synchronous telemental health services during the COVID-19 pandemic (published between March 2020 and June 2023), which observed mostly mixed or nonsignificant associations [28]. Only some initial indications were found for the relationship of female sex, younger age, and lower psychological symptom severity with a higher probability of patient use. No study from Germany exploring determinants of patient use was included in this review [28].

More recent studies with samples from the United States and Canada also considered mostly synchronous services and highlighted the association of female gender, younger age, higher education, higher socioeconomic status, urban residency, speaking English at home, nonrefugee status, lower-risk chief complaints, better self-rated health, caregiver status, access to family physician, and higher eHealth literacy with higher probability of telemental health service use [29-32]. In a sample from South Korea, associations of female gender, older age, previous hospitalization, higher number of outpatient visits, comorbidity, and more severe diagnoses with higher odds of telemental health services use were observed, which partly contrasts with previous findings [33]. Very few recent studies with German samples exist, which identified male gender, younger age, higher education, having acute or residual symptoms, performance and effort expectancy, and electronic literacy as determinants of greater intention to or actual use of the services [34-37].

### Objective and Relevance

Determinants of telemental health service use are scarcely investigated. Particularly, research focusing on determinants of patient use of asynchronous services and the postpandemic context is almost completely missing. Therefore, the aim of this study was to identify determinants of patient use of telemental health services since the onset of the COVID-19 pandemic and in the last 4 weeks. We will focus on a representative sample from Germany, as the German context has been understudied thus far, despite the high potential of telemental health services in this country.

We will focus on key determinants anticipated to be associated with patient use of telemental health services, based on existing literature and theory:

1. Socioeconomic factors: factors such as gender, age, education, or living area were repeatedly linked with telemental health service use in previous studies (eg, [28,29]). According to the Unified Theory of Acceptance and Use of Technology (UTAUT) [38], gender and age may be influential factors in shaping the behavioral intention to use telemedicine.
2. Access factors: previous studies have identified limited broadband internet access as a major barrier to telemental health service use [39,40]. While insurance status has been associated with health care use in Germany (such as increased general physician visits among the statutory insured [41]), the association of insurance status with telemental health service use requires further investigation.
3. Health factors: while higher severity of mental illness was associated with lower probability of telemental health service use in past research [28-30], physical health limitations were linked to higher use of telemedicine services (eg, [42,43]).
4. Psychosocial factors: associations of psychosocial characteristics with health care use (such as loneliness with higher health care use [44]) call for further investigation in telemental health services. The psychosocial UTAUT dimensions and their proven association with telemedicine use require further evaluation in the German mental health context.
5. COVID-19–related factors: COVID-19–related fear or preventive behaviors were found to be associated with telemedicine use in previous studies [42,45], which may also be applicable for telemental health care.
6. Personality: higher levels of agreeableness, extraversion, openness to experience, and particularly higher neuroticism, were associated with increased health care use [46], which was also confirmed in single studies on telemental health services [47-49].
7. Provider and service factors: beyond patient characteristics, patient use of telemental health services may be facilitated by provider attributes or service properties [50].

Telemental health services represent a valuable solution to some of the major challenges in the current provision of mental health care. Consequently, increasing the use of the services should be prioritized. Examining these determinants of patient use of telemental health services may help to uncover disparities in use and access among mental health patients, identify specific target groups and those needing additional support, and highlight potential facilitators and barriers to using the services. This knowledge may ultimately enhance the widespread use of future services. In response to the COVID-19 pandemic, telemental health services were implemented globally [19,20]. While synchronous telemental health services were used on a wide scale during this period, asynchronous service use lagged behind [20]. Expanding knowledge on the determinants of patient use of asynchronous telemental health services may help to realize the potential of the services for mental health patients and contribute to a more comprehensive understanding of telemental health service users. While telemental health services were widely used during the pandemic, the use behavior of mental health patients in the postpandemic context remains to be explored. As severe restrictions disappeared and extensive in-person visit availability returned, determinants of patient use may vary and are of particular interest for future service use.

## Methods

### Sample

We created an online questionnaire, which was pretested in November 2023 (n=13). After pretesting, minor refinements were made to the questionnaire, and the data collection was carried out between December 1 and December 15, 2023. Participants were recruited by the market research firm Bilendi, which is an ISO 20252:2019-certified online sample provider. We collected cross-sectional data from the general adult population in Germany (18‐74 years) and only included individuals who had been using mental health services since March 2020. In addition, participants were recruited based on predetermined quotas for gender and age, reflecting mental health service use rates in Germany [51,52]. In total, 2178 individuals participated in the online survey.

### Ethical Considerations

The incentive system employed by Bilendi is based on a point system or payment of cash. For the purpose of our survey, participants received a real-time cash payment upon successful completion of the questionnaire, with amounts ranging from €0.90 to €1.80 (conversion rate: US $1=€0.924 in 2023). Panel members are able to opt out of the panel at all times. Anonymized data were provided by Bilendi after the data collection was completed. All participants provided informed consent, and the study was approved by the Local Psychological Ethics Committee of the University Medical Center Hamburg-Eppendorf (LPEK-0683).

### Dependent Variable

Patient use of telemental health services was assessed with a single item that inquired whether individuals had used telemental health services as part of their mental health treatment since March 2020. In March 2020, the World Health Organization officially declared COVID-19 a pandemic [53]. During that time, multiple measures to prevent spreading of the virus were implemented, also in Germany. This included extensive changes from in-person health service delivery to remote service forms [23]. Therefore, this date served as a starting point for our telemental health service use measurement. Furthermore, past users of telemental health services were asked to report the frequency of their telemental health service use in the last 4 weeks. Participants who reported using the services at least once in the last 4 weeks were categorized as current users (see Table S1 in Multimedia Appendix 1 for detailed overview of the included variables and their measurement).

### Independent Variables

The association of various determinants with patient use and current use of telemental health services was tested. The determinants were chosen based on theoretical considerations, past findings, and research gaps [28,34-37]. Consequently, we included socioeconomic, access, health, COVID-19–related, psychosocial, and personality factors as well as provider characteristics as determinants. Regarding the current use of telemental health services, we also examined its relationship with service factors (see Table S1 in Multimedia Appendix 1 for detailed overview of the included variables and their measurement).

A number of socioeconomic factors were observed. This included gender (male, female, and diverse or intersex), age, employment status (unemployed, full-time employed, part-time employed, and other), area lived in (urban, mostly urban, and rural), living situation (living with a partner in the same household, living with a partner without a common household, widowed or partner deceased, and single or divorced), migration background (yes or no), and having children or grandchildren (yes or no). The International Standard Classification of Education 1997 (ISCED-97) [54] was used to classify the participants’ educational level. The ISCED-97 levels were summarized into 3 groups, indicating a low (ISCED levels 0‐2), medium (ISCED levels 3 and 4), or high (ISCED levels 5 and 6) educational level. In addition, the monthly household net income was categorized into tertiles (low, medium, and high) using 13 given income categories (1=less than €500, 2=€500 to under €1000, 3=€1000 to under €1500, 4=€1500 to under €2000, 5=€2000 to under €2500, 6=€2500 to under €3000, 7=€3000 to under €3500, 8=€3500 to under €4000, 9=€4000 to under €4500, 10=€4500 to under €5000, 11=€5000 to under €6000, 12=€6000 to under €8000, and 13=€8000 or higher; conversion rate: US $1=€0.924 in 2023). This assessment of household income is consistent with other large surveys, including the German Health Interview and Examination Survey for Adults (DEGS) [55] or the Hamburg City Health Survey [56].

We controlled for differences in access factors. Since differences in regulations and complexity regarding the use of telemedicine exist for the different insurance types in Germany, this factor was considered (statutory health insurance or private health insurance). Furthermore, the internet quality at the patient’s home was assessed (fast and stable; fast, but not stable; stable, but not fast; and neither fast nor stable or no internet connection at home).

In addition, health factors were examined. This included depressive and anxiety symptoms. Depressive symptoms were measured using the Patient Health Questionnaire-9 (PHQ-9) [57]. The PHQ-9 is a well-established self-report measure of depression severity in the last two weeks, which consists of 9 items with a sum score ranging from 0 to 27 (higher values indicate more severe depressive symptoms). The German version of the PHQ-9 was found to be highly reliable (Cronbach α=0.88) and valid (sensitivity=95% and specificity=86%) [58]. In our sample, the Cronbach α of the scale was .88 and McDonald ω was .89. Anxiety symptoms were assessed with the widely used Generalized Anxiety Disorder Scale-7 (GAD-7) [59]. The GAD-7 comprises 7 items measuring anxiety symptoms in the last 2 weeks (sum score range 0‐21, higher values indicate more severe anxiety symptoms). The German version of the GAD-7 has good reliability (Cronbach α=0.89) and validity [60]. For our sample, Cronbach α and McDonald ω were both 0.90. In addition, we assessed the presence of chronic physical illnesses (yes or no) as well as self-rated health using a 5-point Likert scale (ranging from 1=very bad to 5=very good).

We also examined COVID-19–related factors, including COVID-19 vaccination status (yes or no) and fear of COVID-19. Fear of COVID-19 was assessed with the 7-item Fear of COVID-19 Scale (FCV-19S) [61]. Higher FCV-19S scores indicate greater fear of COVID-19 (sum score range 7‐35). The German version of the FCV-19S was found to exhibit good psychometric properties [62]. The scales’ Cronbach α and McDonald ω were both 0.92 in our sample.

We observed several psychosocial determinants. The 6-item De Jong Gierveld Loneliness Scale [63] was used to measure loneliness. The scales’ mean score ranges between 0=absence of loneliness and 11=complete loneliness. The scale was found to measure loneliness in a valid and reliable way, and reached a Cronbach α of 0.80 and McDonald ω of 0.79 in our sample. We also examined perceived social support by family and friends using the 6-item Lubben Social Network Scale (LSNS-6) [64]. The sum score of the LSNS-6 ranges between 0 and 30 (higher values indicate greater perceived social support). The good psychometric properties of the German version of the LSNS-6 were evaluated in the past [64]. In our sample, Cronbach α was 0.85 and McDonald ω was 0.82. In addition, we observed life satisfaction using the 5-item Satisfaction with Life Scale (SWLS) [65]. The sum score for the SWLS ranges between 5=extremely dissatisfied and 35=extremely satisfied. The German version of the SWLS was found to be a valid and reliable measure of life satisfaction [66]. In our sample, the SWLS had a Cronbach α and McDonald ω of 0.91. Furthermore, we included self-efficacy as determinant and assessed it with the 3-item Short Scale for Measuring General Self-efficacy Beliefs (Allgemeine Selbstwirksamkeit Kurzskala [ASKU]) [67]. The ASKU mean score ranges between 1 and 5 (higher scores indicate greater self-efficacy). We used the German version of the ASKU, which has favorable psychometric properties [67] and had a Cronbach α and McDonald ω of 0.89 in our study. Furthermore, we assessed the patient’s attitude towards telemental health services using the Unified Theory of Acceptance and Use of Technology-Patient version questionnaire (UTAUT-P) [68], which shows adequate psychometric properties [68]. Also in our sample, the scale shows a Cronbach α and McDonald ω of 0.87. The UTAUT-P consists of 14 items (sum score range 14‐70, higher values indicate more positive attitudes), which examine 4 factors, including the patients’ therapy quality expectancy, convenience, ease of use, and pressure from others. The scale was designed to explore patient attitudes toward telepsychotherapy in clinical practice and research and is based on the well-established framework of the UTAUT [38]. So far, no validated German translation of the UTAUT-P exists. Consequently, a German translation of the UTAUT-P was created by 2 specialized translators from the professional translation agency tolingo (translation carried out by the first and editing by a second specialized translator; ISO 17100-certified). Considering existing guidelines for the cross-cultural adaptation of self-report measures [69], we made additional refinements to the translation to improve its semantic, idiomatic, experiential, and conceptual equivalence before pretesting the questionnaire.

To test for the association of personality with patient use of telemental health services, we included the Big Five Inventory–Socio-Economic Panel (BFI-S) [70] in our online survey. The BFI-S is based on the 44-item Big Five Inventory [71] and consists of 15 items measuring conscientiousness, extraversion, openness, agreeableness, and neuroticism (each with 3 items). The scale was developed in the context of the Socio-Economic Panel and was found to have mostly acceptable psychometric properties [72]. In our sample, Cronbach α and McDonald ω for the big five personality dimensions ranged between 0.51 (agreeableness) and 0.77 (extraversion).

We additionally asked participants to describe the characteristics of their providers. Similar to former telemedicine research [73,74], we examined provider characteristics through patient ratings. The provider’s attitude toward telemental health services was measured using a single item asking the patients whether they agree that their mental health provider has a positive and open attitude toward telemental health services (ranging from 1=strongly disagree to 5=strongly agree). In addition, provider skills were explored using a single item asking patients whether they agree that their mental health provider has the necessary skills and competencies to use telemental health services without any problems (ranging from 1=strongly disagree to 5=strongly agree).

Finally, we tested for associations of service factors with the current use of telemental health services. We asked participants whether they use telemental health services to avoid stigmatization, for example, to avoid meeting familiar people in mental health facilities (ranging from 1=not true at all to 5=completely true). Furthermore, the level of convenience, which telemental health services offer, was observed (yes, shorter waiting times and easier scheduling of first appointment; yes, shorter waiting times; yes, easier scheduling of first appointment; and no).

### Statistical Analysis

First, descriptive sample characteristics were computed. Second, multiple logistic regressions were performed to explore the association of the determinants with patient use. Previously, model assumptions for logistic regressions were verified (eg, absence of multicollinearity). The first regression model examined associations with patient use of telemental health services since the onset of the pandemic and included all independent variables except service factors. Furthermore, we performed additional logistic regression analyses for this outcome (patient use since the pandemic) with subgroups for different telemental health service types (video, telephone, and asynchronous services) and for the two most prevalent psychiatric diagnoses in Germany (anxiety and affective disorder [75]). The second regression model investigated associations with current use of telemental health services, also including service factors as independent variables. All independent variables were included simultaneously in each of the regression models. Given that a small number of missing values were present in one independent variable (monthly household net income: 4.4% missing), listwise deletion was used to address this issue. Notably, all other variables had no missing values. Statistical significance was considered at an alpha level of *P*<.05. Stata (version 18.0; StataCorp) [76] was used for the statistical analyses. Stata’s “alpha” command and “omegacoef” tool [77] were used to calculate Cronbach α and McDonald ω for all included scales.

## Results

### Sample Characteristics

The descriptive characteristics of all included variables are presented in Table 1. The analytic sample included 2082 individuals, of which 40% (833/2082) were men, 59.7% (1,243/2082) women, and 0.3% (6/2082) diverse or intersex. The sample included individuals from the general adult population in Germany (18‐74 years), with a mean age of 45.7 (SD 11.8) years. Telemental health service use since the onset of the pandemic was reported by 44.5% (926/2082) of the sample. Among all participants, 23.2% (484/2082) had used video services, 20.6% (429/2082) telephone services, and 12.4% (285/2082) asynchronous services. Furthermore, 62.3% (560/899) of the telemental health service users in our analytic sample were classified as current users.

**Table 1.** Sample characteristics (N=2082).

Variables	Values
Reported use of telemental health services	
Since the onset of the COVID-19 pandemic, n (%)	
Yes	926 (44.5)
No	1156 (55.5)
Video services, n (%)	
Yes	484 (23.2)
No	1598 (76.8)
Telephone services, n (%)	
Yes	429 (20.6)
No	1653 (79.4)
Asynchronous services, n (%)	
Yes	285 (12.4)
No	1824 (87.6)
Current use among past users (in the last 4 weeks), n (%)	
Yes	560 (62.3)
No	339 (37.7)
Socioeconomic factors	
Gender, n (%)	
Men	833 (40.0)
Women	1,243 (59.7)
Diverse or intersex	6 (0.3)
Age (range 18‐74), mean (SD)	45.7 (11.8)
Educational level^a^, n (%)	
Low educational level (ISCED 0‐2)	242 (11.6)
Medium educational level (ISCED 3‐4)	1,043 (50.1)
High educational level (ISCED 5‐6)	797 (38.3)
Employment status, n (%)	
Unemployed	709 (34.1)
Full-time employed	830 (39.9)
Part-time employed	387 (18.6)
Other	156 (7.5)
Monthly household net income (€)^b^, n (%)	
Low income (0 to under 2000)	804 (38.6)
Medium income (2000 to under 3500)	683 (32.8)
High income (3500 or higher)	595 (28.6)
Area lived in, n (%)	
Urban	1004 (48.2)
Mostly urban	781 (37.5)
Rural	297 (14.3)
Living situation, n (%)	
Living with partner in the same household	1123 (53.9)
Living with partner without a common household	114 (5.5)
Partner deceased or widowed	58 (2.8)
Single or divorced	787 (37.8)
Migration background, n (%)	
Yes	262 (12.6)
No	1820 (87.4)
Have children, n (%)	
Yes	1156 (55.5)
No	926 (44.5)
Have grandchildren, n (%)	
Yes	334 (16.0)
No	1748 (84.0)
Access factors	
Insurance type, n (%)	
Statutory health insurance	1946 (93.5)
Private health insurance	136 (6.5)
Internet connection quality at home, n (%)	
Fast and stable	1420 (68.2)
Fast, but not stable	273 (13.1)
Stable, but not fast	227 (10.9)
Neither fast nor stable or no internet connection at home	162 (7.8)
Health factors	
Depressive symptoms^c^ (range 0‐27), mean (SD)	12.2 (6.3)
Anxiety symptoms^d^ (range 0‐21), mean (SD)	9.7 (5.4)
Presence of at least 1 chronic physical illness, n (%)	
Yes	1021 (49.0)
No	1061 (51.0)
Self-rated health (range 1‐5), mean (SD)	2.9 (0.9)
COVID-19–related factors	
Received COVID-19 vaccination, n (%)	
Yes	1828 (87.8)
No	254 (12.2)
Fear of COVID-19^e^ (range 7‐35), mean (SD)	13.4 (6.5)
Psychosocial factors	
Loneliness^f^ (mean score range 1‐14), mean (SD)	2.6 (0.7)
Perceived social support by family and friends^g^ (range 0‐30), mean (SD)	11.7 (5.7)
Life satisfaction^h^ (range 5‐35), mean (SD)	17.3 (7.2)
Self-efficacy^i^ (mean score range 1‐5), mean (SD)	3.3 (0.9)
Attitude towards telemental health services^j^ (range 14‐70), mean (SD)	43.2 (10.1)
Personality^k^	
Conscientiousness (range 3‐21), mean (SD)	15.7 (3.4)
Extraversion (range 3‐21), mean (SD)	12.3 (4.1)
Agreeableness (range 3‐21), mean (SD)	15.1 (3.1)
Openness (range 3‐21), mean (SD)	14.1 (3.9)
Neuroticism (range 3‐21), mean (SD)	14.9 (3.7)
Provider characteristics	
Provider attitude toward telemental health services (range 1‐5), mean (SD)	3.1 (1.1)
Provider skills for using telemental health services (range 1‐5), mean (SD)	3.4 (1.1)
Service factors	
Use of the services to avoid stigmatization (range 1‐5), mean (SD)	2.0 (1.2)
Higher convenience of telemental health services, n (%)	
Yes, shorter waiting times and easier scheduling of first appointment	201 (21.7)
Yes, shorter waiting times	237 (25.5)
Yes, easier scheduling of first appointment	78 (8.4)
No	412 (44.4)

a The educational level was measured using the International Standard Classification of Education 1997 (ISCED-97). Higher values indicate a higher educational level.

b conversion rate: US $1=€0.924 in 2023,

c Depressive symptoms were measured using the Patient Health Questionnaire-9 (PHQ-9). Higher values indicate higher depressive symptoms.

d Anxiety symptoms were measured using the Generalized Anxiety Disorder Scale-7 (GAD-7). Higher values indicate higher anxiety symptoms.

e Fear of COVID-19 was measured using the Fear of COVID-19 Scale (FCV-19S). Higher values indicate higher fear of COVID-19.

f Loneliness was measured using the De Jong Gierveld Loneliness Scale. Higher values indicate higher loneliness.

g Perceived social support by family and friends was measured using the Lubben Social Network Scale-6 (LSNS-6). Higher values indicate higher perceived social support by family and friends.

h Life satisfaction was measured using the Satisfaction with Life Scale (SWLS). Higher values indicate higher life satisfaction.

i General self-efficacy was measured using the Short Scale for Measuring General Self-efficacy Beliefs (Allgemeine Selbstwirksamkeit Kurzskala [ASKU]). Higher values indicate higher self-efficacy.

j Attitude toward telemental health services was measured using the Unified Theory of Acceptance and Use of Technology-Patient version (UTAUT-P). Higher values indicate more positive attitudes toward telemental health services.

k Personality was measured using the Big Five Inventory–Socio-Economic Panel (BFI-S). Higher values indicate higher levels of the different personality traits.

### Regression Analyses

#### Patient Use Since the Onset of the COVID-19 Pandemic

**Figure 1.**
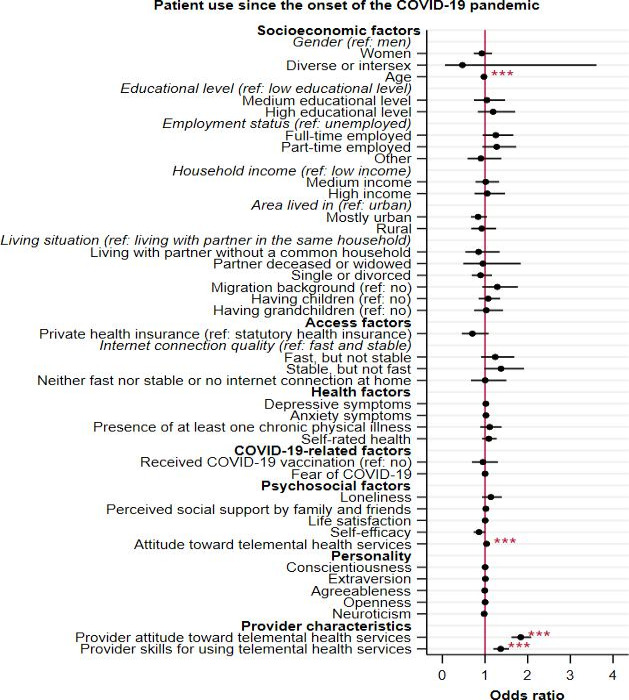
Results of logistic regression for determinants of telemental health service use since the onset of the COVID-19 pandemic among the total sample (N=2082). Odds ratios with 95% CIs are presented. ref: reference category. *** *P*<.001.

In Figure 1, results of the multiple logistic regression for determinants of telemental health service use since the onset of the pandemic are summarized (see Table S1 in Multimedia Appendix 2 for detailed results). Patient use of telemental health services was significantly associated with younger age (OR 0.97, 95% CI 0.96‐0.98; *P*<.001), a positive patient attitude towards telemental health services (OR 1.04, 95% CI 1.02‐1.05; *P*<.001), a positive provider attitude towards telemental health services (OR 1.84, 95% CI 1.62‐2.08; *P*<.001), and higher provider skills for using the services (OR 1.37, 95% CI 1.20‐1.56; *P*<.001).

When computing further subgroup analyses for this outcome for different service types (video, telephone, and asynchronous services) and psychiatric diagnoses (anxiety and affective disorder), these relationships also reached statistical significance. However, additional positive associations of migration background, full-time or part-time employment, higher loneliness, higher perceived social support by family and friends, and greater fear of COVID-19 with patient use of telemental health services were found in single models. Additional negative associations of being a woman, being single or divorced, having private health insurance, higher self-efficacy, and higher neuroticism with patient use were found in some models (see Tables S1-S5 in Multimedia Appendix 3 for detailed results).

#### Patient Use in the Last 4 Weeks

**Figure 2.**
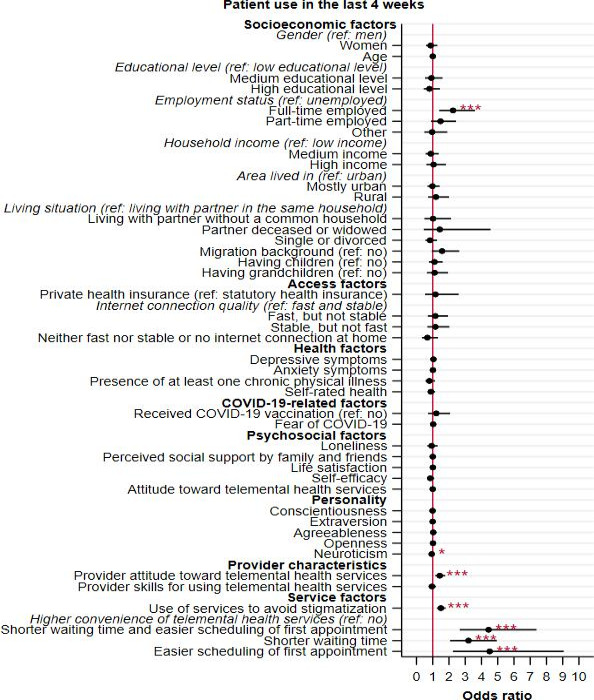
Results of logistic regression for determinants of current use of telemental health services among past users (n=899*).* Odds ratios with 95% Cls are presented. ref: reference category. * *P*<.05, ***P<.001.

When considering the current use of telemental health services, some significant relationships with the determinants were observed (see Figure 2). Multiple logistic regression revealed positive associations of full-time employment (OR 2.25, 95% CI 1.40‐3.60; *P*<.001), lower levels of neuroticism (OR 0.94, 95% CI 0.88‐1.00; *P*=.046), and a positive provider attitude towards the services (OR 1.43, 95% CI 1.16‐1.77; *P*<.001) with the current use of telemental health services. Furthermore, service factors were exclusively included in this model and higher avoidance of stigmatization (OR 1.50, 95% CI 1.26‐1.80; *P*<.001), shorter waiting times and easier scheduling of the first appointment (OR 4.44, 95% CI 2.67‐7.38; *P*<.001), as well as shorter waiting times (OR 3.20, 95%CI 2.07‐4.94; *P*<.001) and easier scheduling (OR 4.51, 95% CI 2.24‐9.06; *P*<.001) individually, were positively associated with the current use of telemental health services (see Table S2 in Multimedia Appendix 2 for detailed results).

## Discussion

### Principal Findings

In this study, we examined determinants of use (since the onset of the COVID-19 pandemic) and current use (in the last 4 weeks) of telemental health services in a representative sample of mental health care users from Germany (in terms of gender and age group). Overall, we found that telemental health service use was positively associated with younger age, a more positive patient attitude toward telemental health services, a more positive provider attitude toward using the services and higher provider skills for using the services. These findings were supported by additional subgroup analyses for service type (video, telephone, and asynchronous services) and psychiatric diagnoses (anxiety and affective disorder). When exclusively looking at current use of telemental health services, positive associations with full-time employment, lower neuroticism, a more positive provider attitude toward the services, use of the services to avoid stigmatization, long waiting times, and inconvenient scheduling were observed. We examined a wide variety of determinants, which have been rarely studied in the past, and therefore, our study provides novel insights into the use behavior of mental health patients regarding telemental health services.

### Relation to Previous Research

When observing the association of socioeconomic characteristics with telemental health service use since the pandemic, mostly nonsignificant associations were found in our sample. This may suggest that telemental health service use is not linked to socioeconomic differences in Germany. No associations of socioeconomic characteristics with remote health consultation use were also found in the German SHARE sample [42]. In contrast, other telemedicine research from Germany found associations of male sex, younger age, higher or lower education, living with a partner in the same household, and having children younger than 18 years with telemedicine use during the pandemic [35,37,43,78-80]. These mixed findings call for further exploration of socioeconomic disparities in telemedicine use particularly focusing on mental health patients.

Nevertheless, we found a significant association of younger age with higher likelihood of telemental health service use in our sample. Previous studies focusing on the German as well as the international context also observed this relationship [28,29,35-37]. This finding may highlight the growing grey digital divide in digital health care, which calls for action in practice and research to prevent the exclusion of older patients in remote health care [81]. Regarding current telemental health services use, only full-time employment status was associated with a higher likelihood of patient use of the services in the last 4 weeks. Full-time employed individuals may continue to value the convenience and travel time savings associated with remote services, even beyond the pandemic. In addition, full-time employed individuals may be more frequently exposed to digital devices, resulting in fewer barriers to telemental health service use. Accordingly, being employed was associated with digital health literacy in the past [82]. More studies are required to examine this relationship in postpandemic circumstances.

None of the included access factors were significantly associated with telemental health service use in our sample. The majority of mental health patients in Germany seems to have access to telemental health services, regardless of variations in internet connection quality at home or differences in individual insurance regulations. This is in line with previous international studies, which also observed no relationship between insurance status and patient use of synchronous telemental health services [83-85]. Nevertheless, disparities in access to broadband internet have been identified as a main barrier to telemedicine use in multiple international studies [39,40], which should be considered in the global context. For instance, Curtis et al [86] examined a large sample of American households and found that broadband internet access was substantially lower in rural households, racial or ethnic minority households, households with no health insurance, less educated and lower-income household.

Surprisingly, neither mental nor physical health factors had a relationship with telemental health service use in our sample. Therefore, telemental health services seem to be accessible to various patient groups. The strengths of remote services may benefit both, patients with good mental or physical health and those with severe mental or physical illness. While healthier patients may value the increased accessibility and convenience of the services in their everyday life (eg, reduced travel time and more flexibility), patients with severe health limitations may appreciate lower barriers to using remote services (eg, no travelling to appointments required and greater comfort at home). However, past findings regarding health determinants are mixed. International studies found significant associations of higher psychological symptom severity with smaller probability of telemental health service use [28-30]. Nevertheless, bad physical and mental health was associated with a higher frequency or probability of telemental health service use in recent studies [29,33,87-90]. Likewise, a study observing a German sample of adults affected by depression found that patients with acute or residual depressive symptoms were more likely to use telemental health services compared to individuals who were symptom-free [36]. More research is needed to clarify the relationship of physical and mental health with telemental health service use, particularly in German samples.

COVID-19–related determinants were not significantly associated with service use in our sample. This is in contrast to research observing significant associations of COVID-19–related challenges or fear with general telemedicine use in Germany during the pandemic [42,45,80,91]. This might be attributed to the later time period during which our data were collected. Throughout the course of the pandemic, fear of COVID-19 has decreased [92], which may have also resulted in a reduction of its associated telemedicine use. In addition, our sample included only mental health patients, who might have felt less threatened by COVID-19 compared to patients with serious physical illnesses, such as cancer or chronic respiratory diseases.

Most of the included psychosocial characteristics were not associated with patient use of telemental health services. Psychosocial factors such as loneliness or life satisfaction might be less influential compared to provider characteristics and service factors, which have a more immediate and direct influence on the patients’ decision to use telemental health services. Nevertheless, significant associations with the patients’ attitude towards telemental health services were observed in our study. The UTAUT dimensions, which were used to measure the patients’ attitude towards the services, have previously been associated with general telemedicine use [93] and also appear to act as a major determinant of patient use of telemental health services. Higher expected therapy quality, convenience, ease of use, and pressure from others, which were measured by the UTAUT-P, might increase patient motivation and reduce resistance to using the services. However, current use of telemental health services was not significantly associated with the patient’s UTAUT scores. Therefore, the UTAUT dimensions may play an important role in the initial use of telemental health services, while other factors may be more critical for the continued use of the services (eg, service factors or personal experience). Accordingly, only single UTAUT dimensions (ie, performance expectancy, social influence) were associated with continued use (intention) in previous telemedicine studies [94,95]. Future studies are required for further examination of this relationship.

None of the examined personality traits were associated with telemental health service use, which suggests that the patients’ use behavior is not majorly affected by their personality. However, current use of the services was associated with lower levels of neuroticism. This is surprising since previous research on telemental health service use and engagement examined a positive relationship with higher levels of neuroticism [47-49,96]. The previous studies argue that individuals with higher neuroticism perceive greater disease threat and are engaging in more favorable health behaviors [47,49,96], which may contribute to higher telemental health service use. A possible explanation for this contrast might be that patients with high neuroticism prefer in-person visits due to a higher need for emotional support. In the postpandemic period, when current use was assessed in our study, the availability of in-person visits returned and may have become the preferred option. Other prepandemic studies observed nonsignificant associations of neuroticism with the intention to use or actual use of telemental health services [97-99]. These mixed findings highlight the need for further research on the association of personality with patient use of telemental health services, particularly for neuroticism and the postpandemic context.

A positive provider attitude toward telemental health services was positively associated with patient use of telemental health services. Thus, mental health care providers play a crucial role in facilitating patient use and have accordingly been identified as gatekeepers to telemental health service use in numerous studies [50]. Even though providers were found to have positive attitudes toward telemental health services, multiple barriers to service use still exist [100]. For example, German mental health professionals reported legal aspects, data protection regulations, lack of personalization, safety concerns, the quality of technical equipment, and lack of information on evidence and regulations as main barriers in a recent study [101]. Therefore, providing guidelines, resources, and support for providers may be beneficial in the future to enable widespread use of the services (eg, [50]). Likewise, better provider skills for using the services were associated with increased patient use of telemental health services in our study, which further underscores the need for provider support to increase patient use of the services.

Regarding current telemental health service use, we additionally examined associations with service factors. Using the services to avoid stigmatization (eg, to avoid meeting familiar people in mental health facilities) was associated with higher probability of current use. Recent reviews also stated that telemental health services can help to overcome the barrier of stigma associated with seeking mental health treatment [102,103]. Therefore, telemental health services may play a crucial role in facilitating use of mental health care. Furthermore, shorter waiting times and easier scheduling of appointments via telemental health services were associated with current use, which further highlights the services’ potential for patients. Access issues such as long waiting times and lengthy scheduling processes are a barrier to mental health treatment not only in Germany [25], but also globally [9,12]. Telemental health services are a valuable resource in tackling this issue.

### Strengths and Limitations

We used data from a representative sample of mental health patients in Germany with respect to gender and age [51,52]. Thus, the use behavior of a wide range of mental health patients was inspected. Furthermore, we observed a number of independent variables and gained new insights into their association with patient use of telemental health services. More specifically, our study adds new knowledge to the current literature, regarding socioeconomic, access, health, COVID-19–related, psychosocial, and service factors as well as personality, and provider characteristics. Furthermore, we considered different service types, psychiatric diagnoses, and the postpandemic context, areas for which the existing research is limited. The included variables were measured using validated and reliable questionnaires, which adds to the credibility of our findings.

However, some limitations should be noted. We observed cross-sectional data, which limits conclusions regarding causality and longitudinal stability of the observed associations. Consequently, future longitudinal studies are needed to secure our findings. Since we conducted an online survey, our sample of mental health patients may be more likely to have access to and skills for using online services, which might have caused some selection bias. Nevertheless, the great majority of the German population has access to a sufficient internet connection. Furthermore, since we only considered anxiety and affective disorders in subgroup analyses, it remains to be explored whether determinants of patient use of telemental health service vary across other specific samples, such as individuals with other serious mental illnesses (eg, schizophrenia or bipolar disorder) or those in forensic settings. Finally, we only included patients from Germany, which restricts the generalizability of the findings to other countries. Future international research is required to draw conclusions regarding country-specific health care systems.

### Conclusion

Expanding the knowledge on determinants of telemental health service use is essential to increase accessibility to and widespread use of the services. Patient characteristics, such as younger age or positive attitudes toward the services, were associated with increased service use, which may help to define target groups for telemental health services. Nevertheless, efforts are needed to also reach older patients and positively influence patient attitudes toward the services to increase overall service use. Future studies focusing on nonusers might contribute to identifying crucial barriers and misconceptions regarding telemental health services. This is increasingly important in the light of the potential and ongoing development of the digital mental health care landscape. Beyond the examined services, emerging technologies such as virtual reality or artificial intelligence are gaining prominence in the treatment of mental illness, and these innovations may play an increasing role in mental health care [104].

Apart from patient characteristics, provider skills for using and attitude toward the services as well as service factors (reduced stigmatization and higher convenience) showed significant relationships with the outcome. Therefore, providers play an essential role in facilitating patient use of telemental health services. Service awareness among providers should be promoted, along with offers of support and training. Furthermore, telemental health services represent a possible solution to nonuse of mental health services due to fear of stigmatization and inconvenience of service use (eg, long waiting times), which was supported by our findings. This is essential for addressing unmet needs in the provision of mental health care and should be actively promoted to both patients and providers.

## Supplementary material

10.2196/70925 Multimedia Appendix 1 Included variables and their measurement.

10.2196/70925 Multimedia Appendix 2 Detailed results of logistic regression for determinants of telemental health service use.

10.2196/70925 Multimedia Appendix 3 Detailed results of logistic regressions for determinants of telemental health service use for different service types and psychiatric diagnoses.

## Abbreviations

ASKU: Allgemeine Selbstwirksamkeit Kurzskala
BFI-S: Big Five Inventory–Socio-Economic Panel
FCV-19S: 7-item Fear of COVID-19 Scale
ISCED-97: International Standard Classification of Education 1997
LSNS-6: 6-item Lubben Social Network Scale
PHQ-9: Patient Health Questionnaire-9
SWLS: Satisfaction with Life Scale
UTAUT: Unified Theory of Acceptance and Use of Technology
UTAUT-P: Unified Theory of Acceptance and Use of Technology-Patient version
