# Supplementary material for: Determinants of Patient Use of Telemental Health Services: Representative Cross-Sectional Survey From Germany
Source: JMIR Ment Health. 2025 Jun 13;12:e70925. doi: 10.2196/70925 (PMC12180686; doi:10.2196/70925)
Supplement: Multimedia Appendix 1 [file mental-v12-e70925-s001.docx]

Table S1. Included variables and their measurement.

| **Variables** | | **Survey Items** | **Response** |
| --- | --- | --- | --- |
| **Patient Use** | Since the onset of the COVID-19 pandemic | Did you use telemental health services as part of your mental health treatment since March 2020? | Yes, no |
|  | Current use | How often do you currently use telemental health services as part of your mental health treatment (i.e., in the last 4 weeks)? | Daily, several times a week, once a week, one to three times a month, less often |
|  | Service Type | Which services did you use? | Video services (online therapy via video consultations), telephone services (therapy over the phone), digital health apps (e.g., Selfapy, Mindable, elona therapy), online intervention/training (e.g., edupression.com, Novego), other |
| **Socioeconomic** | Gender | What is your gender? | Male, female, diverse/intersex |
|  | Age | In which year were you born? | Year of birth |
|  | Education | International Standard Classification of Education 97 (low, medium, high) | |
|  | Employment status | What is your current employment situation? | Full-time employed, part-time employed, unemployed, other |
|  | Household income | What is the average monthly net income of your household? | Less than 500 €, 500 € to under 1,000 €, 1,000 € to under 1,500 €,  1,500 € to under 2,000 €, 2,000 € to under 2,500 €, 2,500 € to under 3,000 €, 3,000 € to under 3,500 €, 3,500 € to under 4,000 €, 4,000 € to under 4,500 €, 4,500 € to under 5,000 €, 5,000 € to under 6,000 €, 6,000 € to under 8,000 €, 8,000 € or higher |
|  | Residential form of partnership | What is your current relationship status? | Living with a partner in the same household, living with a partner without a common household, widowed/partner deceased, single/divorced |
|  | Area lived in | What is the postcode of your current place of residence? | Categorization into urban, mostly urban, and rural according to postcode |
|  | Migration background | Do you have a migrant background?  A person has a migration background if they themselves or at least one of their parents were not born with German citizenship. | Yes, no |
|  | Presence of own (grand)children | Do you have children/grandchildren? | Yes, no |
| **Access** | Insurance type | What is your current insurance? | Statutory health insurance, private health insurance |
|  | Internet connection quality | Do you have a stable (uninterrupted) and fast (smooth, fast loading of content) Internet connection? | Yes, I have a fast and stable internet connection; my internet connection is fast, but not stable; My internet connection is stable, but not fast; No, my internet connection is neither fast nor stable/I do not have an internet connection at home |
| **Health** | Depression | 9-item Patient Health Questionnaire-9 (PHQ-9), scores ranging from 0 to 27, higher values indicate more severe depressive symptoms | |
|  | Anxiety | 7-item Generalized Anxiety Disorder Scale-7 (GAD-7), scores ranging from 0 to 21, higher values indicate more severe anxiety symptoms | |
|  | Physical illness | Do you have at least one chronic physical illness (e.g., diabetes, heart disease)? | Yes, no |
|  | Self-rated health | How would you rate your current state of health? | Very bad, bad, average, good, very good |
| **COVID-19** | Vaccination status | Have you received at least one COVID-19 vaccination? | Yes, no |
|  | Fear | 7-item Fear of COVID-19 Scale (FCV-19S), scores ranging from 7 to 35, higher values indicate greater fear of COVID-19 | |
| **Psychosocial** | Loneliness | 6-item De Jong Gierveld Loneliness Scale, scores ranging from 1 to 4, higher values indicate higher levels of loneliness | |
|  | Social support | 6-item Lubben Social Network Scale (LSNS-6), scores ranging from 1 to 30, higher values indicate greater perceived social support | |
|  | Life satisfaction | 5-item German version of the Satisfaction with Life Scale (SWLS), scores ranging from 1 to 5, higher values indicate greater life satisfaction | |
|  | Self-efficacy | 3-item Short Scale for Measuring General Self-efficacy Beliefs ([Allgemeine Selbstwirksamkeit Kurzskala] ASKU), scores ranging from 1 to 5, higher values indicate greater self-efficacy | |
|  | Attitude toward telemedicine | 14-item Unified Theory of Acceptance and Use of Technology-Patient version questionnaire (UTAUT-P), scores ranging from 17 to 70, higher values indicate more positive attitudes | |
|  | Personality | 15-item Big Five Inventory-SOEP (BFI-S) | |
| **Provider** | Attitude | Now think about your current psychotherapist/psychiatrist/doctor. To what extent do the following statements apply? If you are not currently undergoing mental health treatment, please think of your last psychotherapist/psychiatrist/doctor.  My therapist has a positive and open attitude towards telemental health services (e.g., offers and advertises them). | Strongly disagree, disagree, neither, agree, strongly agree |
|  | Skills | My therapist has the necessary skills to use telemental health services successfully and without problems (e.g., has technical skills and knowledge of the digital programs). | Strongly disagree, disagree, neither, agree, strongly agree |
| **Service** | Stigmatization | Do you use telemental health services as part of your mental health treatment to avoid stigmatization (e.g., to avoid meeting people you know in mental health facilities)? | Not true at all, not quite true, partially true, fairly true, completely true |
|  | Higher Convenience | Do you use telemental health services as part of your mental health treatment because the waiting time for treatment is shorter, or it was easier to make a first appointment than with the usual face-to-face formats? | Yes, shorter waiting times and easier scheduling of first appointment; yes, shorter waiting times; yes, easier scheduling of first appointment; no |
