# Supplementary material for: Determinants of Patient Use of Telemental Health Services: Representative Cross-Sectional Survey From Germany
Source: JMIR Ment Health. 2025 Jun 13;12:e70925. doi: 10.2196/70925 (PMC12180686; doi:10.2196/70925)
Supplement: Multimedia Appendix 2 [file mental-v12-e70925-s002.docx]

Table S1. Results of logistic regression for determinants of telemental health service use since the COVID-19 pandemic among the total sample (n=2,082).

| **Variables** | **Values** |
| --- | --- |
|  |  |
| ***Socioeconomic factors*** |  |
| *Gender (ref: men)* |  |
| Women | 0.93 |
|  | (0.74 - 1.16) |
| Diverse or intersex | 0.47 |
|  | (0.06 - 3.61) |
| Age | 0.97*** |
|  | (0.96 - 0.98) |
| *Educational level (ref: low educational level)* |  |
| Medium educational level | 1.04 |
|  | (0.74 - 1.47) |
| High educational level | 1.19 |
|  | (0.83 - 1.71) |
| *Employment status (ref: unemployed)* |  |
| Full-time employed | 1.25 |
|  | (0.94 - 1.66) |
| Part-time employed | 1.27 |
|  | (0.94 - 1.73) |
| Other | 0.90 |
|  | (0.59 - 1.38) |
| *Household income (ref: low income)* |  |
| Medium income | 1.02 |
|  | (0.78 - 1.33) |
| High income | 1.05 |
|  | (0.75 - 1.47) |
| *Area lived in (ref: urban)* |  |
| Mostly urban | 0.84 |
|  | (0.67 - 1.05) |
| Rural | 0.92 |
|  | (0.68 - 1.26) |
| *Living situation (ref: living with partner in the same household)* |  |
| Living with partner without a common household | 0.85 |
|  | (0.54 - 1.34) |
| Partner deceased or widowed | 0.95 |
|  | (0.49 - 1.84) |
| Single or divorced | 0.90 |
|  | (0.69 - 1.16) |
| Migration background (ref: no) | 1.29 |
|  | (0.94 - 1.77) |
| Having children (ref: no) | 1.07 |
|  | (0.85 - 1.35) |
| Having grandchildren (ref: no) | 1.03 |
|  | (0.74 - 1.42) |
| ***Access factors*** |  |
| Private health insurance (ref: statutory health insurance) | 0.70 |
|  | (0.46 - 1.09) |
| *Internet connection quality at home (ref: fast and stable)* |  |
| Fast, but not stable | 1.24 |
|  | (0.91 - 1.69) |
| Stable, but not fast | 1.37 |
|  | (0.99 - 1.91) |
| Neither fast nor stable or no internet connection at home | 1.00 |
|  | (0.67 - 1.50) |
| ***Health factors*** |  |
| Depressive symptoms | 1.02 |
|  | (0.99 - 1.05) |
| Anxiety symptoms | 1.02 |
|  | (0.99 - 1.06) |
| Presence of at least one chronic physical illness (ref: no) | 1.11 |
|  | (0.89 - 1.39) |
| Self-rated health | 1.09 |
|  | (0.93 - 1.27) |
| ***COVID-19-related factors*** |  |
| Received COVID-19 vaccination (ref: no) | 0.95 |
|  | (0.69 - 1.30) |
| Fear of COVID-19 | 1.00 |
|  | (0.98 - 1.02) |
| ***Psychosocial factors*** |  |
| Loneliness | 1.14 |
|  | (0.93 - 1.39) |
| Perceived social support by family and friends | 1.02 |
|  | (1.00 - 1.04) |
| Life satisfaction | 1.00 |
|  | (0.98 - 1.03) |
| Self-efficacy | 0.86 |
|  | (0.73 - 1.01) |
| Attitude toward telemental health services | 1.04*** |
|  | (1.02 - 1.05) |
| ***Personality*** |  |
| Conscientiousness | 1.00 |
|  | (0.97 - 1.04) |
| Extraversion | 1.01 |
|  | (0.98 - 1.04) |
| Agreeableness | 0.99 |
|  | (0.96 - 1.03) |
| Openness | 1.00 |
|  | (0.97 - 1.03) |
| Neuroticism | 0.98 |
|  | (0.94 - 1.02) |
| ***Provider characteristics*** |  |
| Provider attitude toward telemental health services | 1.84*** |
|  | (1.62 - 2.08) |
| Provider skills for using telemental health services | 1.37*** |
|  | (1.20 - 1.56) |
| Constant | 0.01*** |
|  | (0.00 - 0.06) |
|  |  |
| Observations | 2,082 |
| Pseudo R-squared | 0.210 |

*Note.* Odds Ratios are reported with 95% confidence interval in parentheses. *** p<0.001, ** p<0.01, * p<0.05.

Table S2. Results of logistic regression for determinants of current use of telemental health services among past users (n=899).

| **Variables** | **Values** |
| --- | --- |
|  |  |
| ***Socioeconomic factors*** |  |
| *Gender (ref: men)* |  |
| Women | 0.87 |
|  | (0.59 - 1.29) |
| Diverse or intersex | - |
|  |  |
| Age | 1.00 |
|  | (0.99 - 1.02) |
| *Educational level (ref: low educational level)* |  |
| Medium educational level | 0.92 |
|  | (0.52 - 1.60) |
| High educational level | 0.80 |
|  | (0.44 - 1.44) |
| *Employment status (ref: unemployed)* |  |
| Full-time employed | 2.25*** |
|  | (1.40 - 3.60) |
| Part-time employed | 1.48 |
|  | (0.90 - 2.43) |
| Other | 0.96 |
|  | (0.49 - 1.90) |
| *Household income (ref: low income)* |  |
| Medium income | 0.88 |
|  | (0.56 - 1.37) |
| High income | 1.05 |
|  | (0.61 - 1.81) |
| *Area lived in (ref: urban)* |  |
| Mostly urban | 0.98 |
|  | (0.68 - 1.42) |
| Rural | 1.20 |
|  | (0.72 - 2.00) |
| *Living situation (ref: living with partner in the same household)* |  |
| Living with partner without a common household | 1.02 |
|  | (0.49 - 2.13) |
| Partner deceased or widowed | 1.43 |
|  | (0.45 - 4.56) |
| Single or divorced | 0.84 |
|  | (0.55 - 1.27) |
| Migration background (ref: no) | 1.58 |
|  | (0.94 - 2.63) |
| Having children (ref: no) | 1.11 |
|  | (0.76 - 1.61) |
| Having grandchildren (ref: no) | 1.12 |
|  | (0.64 - 1.94) |
| ***Access factors*** |  |
| Private health insurance (ref: statutory health insurance) | 1.17 |
|  | (0.53 - 2.59) |
| *Internet connection quality at home (ref: fast and stable)* |  |
| Fast, but not stable | 1.16 |
|  | (0.69 - 1.94) |
| Stable, but not fast | 1.16 |
|  | (0.67 - 2.02) |
| Neither fast nor stable or no internet connection at home | 0.67 |
|  | (0.34 - 1.33) |
| ***Health factors*** |  |
| Depressive symptoms | 1.04 |
|  | (0.99 - 1.09) |
| Anxiety symptoms | 1.01 |
|  | (0.96 - 1.07) |
| Presence of at least one chronic physical illness (ref: no) | 0.79 |
|  | (0.55 - 1.14) |
| Self-rated health | 0.89 |
|  | (0.69 - 1.15) |
| ***COVID-19-related factors*** |  |
| Received COVID-19 vaccination (ref: no) | 1.22 |
|  | (0.72 - 2.07) |
| Fear of COVID-19 | 1.03 |
|  | (1.00 - 1.06) |
| ***Psychosocial factors*** |  |
| Loneliness | 0.93 |
|  | (0.67 - 1.30) |
| Perceived social support by family and friends | 1.00 |
|  | (0.97 - 1.04) |
| Life satisfaction | 1.01 |
|  | (0.98 - 1.04) |
| Self-efficacy | 0.85 |
|  | (0.66 - 1.09) |
| Attitude toward telemental health services | 1.00 |
|  | (0.98 - 1.02) |
| ***Personality*** |  |
| Conscientiousness | 0.99 |
|  | (0.94 - 1.05) |
| Extraversion | 1.00 |
|  | (0.95 - 1.05) |
| Agreeableness | 1.03 |
|  | (0.97 - 1.10) |
| Openness | 1.02 |
|  | (0.97 - 1.07) |
| Neuroticism | 0.94* |
|  | (0.88 - 1.00) |
| ***Provider characteristics*** |  |
| Provider attitude toward telemental health services | 1.43*** |
|  | (1.16 - 1.77) |
| Provider skills for using telemental health services | 0.95 |
|  | (0.75 - 1.20) |
| ***Service factors*** |  |
| Use of services to avoid stigmatization | 1.50*** |
|  | (1.26 - 1.80) |
|  |  |
| Higher convenience of telemental health services (ref: no) |  |
| Shorter waiting times and easier scheduling of first appointment | 4.44*** |
|  | (2.67 - 7.38) |
| Shorter waiting times | 3.20*** |
|  | (2.07 - 4.94) |
| Easier scheduling of first appointment | 4.51*** |
|  | (2.24 - 9.06) |
| Constant | 0.11 |
|  | (0.01 - 1.37) |
|  |  |
| Observations | 899 |
| Pseudo R-squared | 0.255 |

*Note.* Odds Ratios are reported with 95% confidence interval in parentheses. *** p<0.001, ** p<0.01, * p<0.05.
