# Supplementary material for: Determinants of Patient Use of Telemental Health Services: Representative Cross-Sectional Survey From Germany
Source: JMIR Ment Health. 2025 Jun 13;12:e70925. doi: 10.2196/70925 (PMC12180686; doi:10.2196/70925)
Supplement: Multimedia Appendix 3 [file mental-v12-e70925-s003.docx]

Table S1. Results of logistic regression for determinants of patient use of video services since the COVID-19 pandemic among the total sample (n=2,082).

| **Variables** | **Values** |
| --- | --- |
|  |  |
| ***Socioeconomic factors*** |  |
| *Gender (ref: men)* |  |
| Women | 0.68** |
|  | (0.52 - 0.90) |
| Diverse or intersex | 0.50 |
|  | (0.04 - 6.53) |
| Age | 0.96*** |
|  | (0.95 - 0.98) |
| *Educational level (ref: low educational level)* |  |
| Medium educational level | 1.24 |
|  | (0.80 - 1.93) |
| High educational level | 1.54 |
|  | (0.98 - 2.42) |
| *Employment status (ref: unemployed)* |  |
| Full-time employed | 1.37 |
|  | (0.96 - 1.95) |
| Part-time employed | 1.42 |
|  | (0.97 - 2.08) |
| Other | 1.14 |
|  | (0.68 - 1.91) |
| *Household income (ref: low income)* |  |
| Medium income | 1.25 |
|  | (0.91 - 1.73) |
| High income | 1.26 |
|  | (0.86 - 1.87) |
| *Area lived in (ref: urban)* |  |
| Mostly urban | 0.92 |
|  | (0.71 - 1.21) |
| Rural | 1.00 |
|  | (0.69 - 1.45) |
| *Living situation (ref: living with partner in the same household)* |  |
| Living with partner without a common household | 1.03 |
|  | (0.61 - 1.75) |
| Partner deceased or widowed | 0.67 |
|  | (0.25 - 1.74) |
| Single or divorced | 0.96 |
|  | (0.70 - 1.31) |
| Migration background (ref: no) | 1.04 |
|  | (0.73 - 1.49) |
| Having children (ref: no) | 1.11 |
|  | (0.84 - 1.47) |
| Having grandchildren (ref: no) | 0.78 |
|  | (0.51 - 1.20) |
| ***Access factors*** |  |
| Private health insurance (ref: statutory health insurance) | 0.74 |
|  | (0.44 - 1.23) |
| *Internet connection quality at home (ref: fast and stable)* |  |
| Fast, but not stable | 1.20 |
|  | (0.83 - 1.73) |
| Stable, but not fast | 1.05 |
|  | (0.69 - 1.60) |
| Neither fast nor stable or no internet connection at home | 0.71 |
|  | (0.39 - 1.28) |
| ***Health factors*** |  |
| Depressive symptoms | 1.01 |
|  | (0.98 - 1.05) |
| Anxiety symptoms | 0.99 |
|  | (0.95 - 1.03) |
| Presence of at least one chronic physical illness (ref: no) | 0.97 |
|  | (0.74 - 1.26) |
| Self-rated health | 1.09 |
|  | (0.91 - 1.31) |
| ***COVID-19-related factors*** |  |
| Received COVID-19 vaccination (ref: no) | 1.03 |
|  | (0.70 - 1.52) |
| Fear of COVID-19 | 1.01 |
|  | (0.99 - 1.03) |
| ***Psychosocial factors*** |  |
| Loneliness | 1.09 |
|  | (0.86 - 1.39) |
| Perceived social support by family and friends | 1.01 |
|  | (0.98 - 1.03) |
| Life satisfaction | 1.00 |
|  | (0.98 - 1.03) |
| Self-efficacy | 0.82* |
|  | (0.68 - 0.99) |
| Attitude toward telemental health services | 1.06*** |
|  | (1.04 - 1.07) |
| ***Personality*** |  |
| Conscientiousness | 0.98 |
|  | (0.94 - 1.02) |
| Extraversion | 1.02 |
|  | (0.99 - 1.06) |
| Agreeableness | 0.99 |
|  | (0.95 - 1.04) |
| Openness | 1.02 |
|  | (0.98 - 1.05) |
| Neuroticism | 0.99 |
|  | (0.95 - 1.04) |
| ***Provider characteristics*** |  |
| Provider attitude toward telemental health services | 1.72*** |
|  | (1.47 - 2.00) |
| Provider skills for using telemental health services | 1.53*** |
|  | (1.29 - 1.82) |
| Constant | 0.00*** |
|  | (0.00 - 0.01) |
|  |  |
| Observations | 2,082 |
| Pseudo R-squared | 0.258 |

*Note.* Odds Ratios are reported with 95% confidence interval in parentheses. *** p<0.001, ** p<0.01, * p<0.05.

Table S2. Results of logistic regression for determinants of patient use of telephone services since the COVID-19 pandemic among the total sample (n=2,082).

| **Variables** | **Values** |
| --- | --- |
|  |  |
| ***Socioeconomic factors*** |  |
| *Gender (ref: men)* |  |
| Women | 0.92 |
|  | (0.71 - 1.20) |
| Diverse or intersex | 0.85 |
|  | (0.08 - 8.71) |
| Age | 0.98* |
|  | (0.97 - 1.00) |
| *Educational level (ref: low educational level)* |  |
| Medium educational level | 1.12 |
|  | (0.75 - 1.68) |
| High educational level | 1.17 |
|  | (0.77 - 1.78) |
| *Employment status (ref: unemployed)* |  |
| Full-time employed | 1.23 |
|  | (0.88 - 1.72) |
| Part-time employed | 1.40 |
|  | (0.98 - 2.01) |
| Other | 0.92 |
|  | (0.55 - 1.55) |
| *Household income (ref: low income)* |  |
| Medium income | 1.02 |
|  | (0.75 - 1.40) |
| High income | 1.08 |
|  | (0.74 - 1.58) |
| *Area lived in (ref: urban)* |  |
| Mostly urban | 1.00 |
|  | (0.77 - 1.30) |
| Rural | 1.31 |
|  | (0.93 - 1.85) |
| *Living situation (ref: living with partner in the same household)* |  |
| Living with partner without a common household | 1.09 |
|  | (0.66 - 1.79) |
| Partner deceased or widowed | 0.85 |
|  | (0.38 - 1.86) |
| Single or divorced | 0.74 |
|  | (0.55 - 1.00) |
| Migration background (ref: no) | 1.41* |
|  | (1.00 - 1.99) |
| Having children (ref: no) | 1.17 |
|  | (0.89 - 1.52) |
| Having grandchildren (ref: no) | 0.91 |
|  | (0.62 - 1.32) |
| ***Access factors*** |  |
| Private health insurance (ref: statutory health insurance) | 0.91 |
|  | (0.56 - 1.49) |
| *Internet connection quality at home (ref: fast and stable)* |  |
| Fast, but not stable | 1.03 |
|  | (0.72 - 1.47) |
| Stable, but not fast | 1.24 |
|  | (0.83 - 1.83) |
| Neither fast nor stable or no internet connection at home | 1.15 |
|  | (0.71 - 1.84) |
| ***Health factors*** |  |
| Depressive symptoms | 1.02 |
|  | (0.99 - 1.06) |
| Anxiety symptoms | 1.00 |
|  | (0.96 - 1.04) |
| Presence of at least one chronic physical illness (ref: no) | 1.08 |
|  | (0.84 - 1.39) |
| Self-rated health | 0.96 |
|  | (0.81 - 1.14) |
| ***COVID-19-related factors*** |  |
| Received COVID-19 vaccination (ref: no) | 0.76 |
|  | (0.53 - 1.08) |
| Fear of COVID-19 | 1.03** |
|  | (1.01 - 1.05) |
| ***Psychosocial factors*** |  |
| Loneliness | 1.14 |
|  | (0.90 - 1.43) |
| Perceived social support by family and friends | 1.01 |
|  | (0.99 - 1.04) |
| Life satisfaction | 0.99 |
|  | (0.96 - 1.01) |
| Self-efficacy | 0.94 |
|  | (0.79 - 1.12) |
| Attitude toward telemental health services | 1.02** |
|  | (1.01 - 1.03) |
| ***Personality*** |  |
| Conscientiousness | 1.01 |
|  | (0.97 - 1.05) |
| Extraversion | 1.01 |
|  | (0.98 - 1.05) |
| Agreeableness | 1.01 |
|  | (0.97 - 1.06) |
| Openness | 1.00 |
|  | (0.97 - 1.04) |
| Neuroticism | 0.98 |
|  | (0.94 - 1.02) |
| ***Provider characteristics*** |  |
| Provider attitude toward telemental health services | 1.49*** |
|  | (1.28 - 1.73) |
| Provider skills for using telemental health services | 1.42*** |
|  | (1.20 - 1.67) |
| Constant | 0.01*** |
|  | (0.00 - 0.03) |
|  |  |
| Observations | 2,082 |
| Pseudo R-squared | 0.140 |

*Note.* Odds Ratios are reported with 95% confidence interval in parentheses. *** p<0.001, ** p<0.01, * p<0.05.

Table S3. Results of logistic regression for determinants of patient use of asynchronous services since the COVID-19 pandemic among the total sample (n=2,082).

| **Variables** | **Values** |
| --- | --- |
|  |  |
| ***Socioeconomic factors*** |  |
| *Gender (ref: men)* |  |
| Women | 0.82 |
|  | (0.59 - 1.13) |
| Diverse or intersex | 1.02 |
|  | (0.07 - 15.09) |
| Age | 0.97*** |
|  | (0.96 - 0.99) |
| *Educational level (ref: low educational level)* |  |
| Medium educational level | 1.20 |
|  | (0.69 - 2.07) |
| High educational level | 1.45 |
|  | (0.83 - 2.53) |
| *Employment status (ref: unemployed)* |  |
| Full-time employed | 2.56*** |
|  | (1.62 - 4.05) |
| Part-time employed | 2.03** |
|  | (1.23 - 3.36) |
| Other | 1.61 |
|  | (0.83 - 3.12) |
| *Household income (ref: low income)* |  |
| Medium income | 1.08 |
|  | (0.72 - 1.61) |
| High income | 1.16 |
|  | (0.73 - 1.83) |
| *Area lived in (ref: urban)* |  |
| Mostly urban | 0.82 |
|  | (0.60 - 1.13) |
| Rural | 0.82 |
|  | (0.52 - 1.29) |
| *Living situation (ref: living with partner in the same household)* |  |
| Living with partner without a common household | 0.74 |
|  | (0.39 - 1.41) |
| Partner deceased or widowed | 0.85 |
|  | (0.30 - 2.39) |
| Single or divorced | 0.63* |
|  | (0.43 - 0.92) |
| Migration background (ref: no) | 0.79 |
|  | (0.51 - 1.21) |
| Having children (ref: no) | 1.20 |
|  | (0.86 - 1.66) |
| Having grandchildren (ref: no) | 0.88 |
|  | (0.54 - 1.46) |
| ***Access factors*** |  |
| Private health insurance (ref: statutory health insurance) | 1.00 |
|  | (0.58 - 1.72) |
| *Internet connection quality at home (ref: fast and stable)* |  |
| Fast, but not stable | 1.04 |
|  | (0.68 - 1.59) |
| Stable, but not fast | 0.60 |
|  | (0.34 - 1.07) |
| Neither fast nor stable or no internet connection at home | 0.56 |
|  | (0.27 - 1.17) |
| ***Health factors*** |  |
| Depressive symptoms | 1.03 |
|  | (0.99 - 1.08) |
| Anxiety symptoms | 1.03 |
|  | (0.98 - 1.08) |
| Presence of at least one chronic physical illness (ref: no) | 1.04 |
|  | (0.76 - 1.42) |
| Self-rated health | 1.12 |
|  | (0.91 - 1.39) |
| ***COVID-19-related factors*** |  |
| Received COVID-19 vaccination (ref: no) | 1.36 |
|  | (0.85 - 2.19) |
| Fear of COVID-19 | 1.02 |
|  | (1.00 - 1.05) |
| ***Psychosocial factors*** |  |
| Loneliness | 1.00 |
|  | (0.76 - 1.33) |
| Perceived social support by family and friends | 0.99 |
|  | (0.96 - 1.02) |
| Life satisfaction | 1.00 |
|  | (0.97 - 1.03) |
| Self-efficacy | 0.98 |
|  | (0.79 - 1.22) |
| Attitude toward telemental health services | 1.03** |
|  | (1.01 - 1.05) |
| ***Personality*** |  |
| Conscientiousness | 0.96 |
|  | (0.91 - 1.01) |
| Extraversion | 0.98 |
|  | (0.94 - 1.02) |
| Agreeableness | 1.03 |
|  | (0.98 - 1.08) |
| Openness | 1.02 |
|  | (0.97 - 1.06) |
| Neuroticism | 0.92** |
|  | (0.88 - 0.97) |
| ***Provider characteristics*** |  |
| Provider attitude toward telemental health services | 1.53*** |
|  | (1.26 - 1.85) |
| Provider skills for using telemental health services | 0.85 |
|  | (0.69 - 1.03) |
| Constant | 0.03** |
|  | (0.00 - 0.28) |
|  |  |
| Observations | 2,082 |
| Pseudo R-squared | 0.166 |

*Note.* Odds Ratios are reported with 95% confidence interval in parentheses. *** p<0.001, ** p<0.01, * p<0.05.

Table S4. Results of logistic regression for determinants of telemental health service use among patients with an anxiety diagnosis since the COVID-19 pandemic (n=983).

| **Variables** | **Values** |
| --- | --- |
|  |  |
| ***Socioeconomic factors*** |  |
| *Gender (ref: men)* |  |
| Women | 0.99 |
|  | (0.69 - 1.40) |
| Diverse or intersex | - |
|  |  |
| Age | 0.97** |
|  | (0.96 - 0.99) |
| *Educational level (ref: low educational level)* |  |
| Medium educational level | 0.94 |
|  | (0.58 - 1.51) |
| High educational level | 1.23 |
|  | (0.74 - 2.05) |
| *Employment status (ref: unemployed)* |  |
| Full-time employed | 1.16 |
|  | (0.76 - 1.78) |
| Part-time employed | 1.24 |
|  | (0.80 - 1.94) |
| Other | 1.17 |
|  | (0.62 - 2.19) |
| *Household income (ref: low income)* |  |
| Medium income | 0.86 |
|  | (0.58 - 1.29) |
| High income | 0.90 |
|  | (0.55 - 1.47) |
| *Area lived in (ref: urban)* |  |
| Mostly urban | 0.89 |
|  | (0.63 - 1.25) |
| Rural | 0.80 |
|  | (0.50 - 1.28) |
| *Living situation (ref: living with partner in the same household)* |  |
| Living with partner without a common household | 0.70 |
|  | (0.35 - 1.41) |
| Partner deceased or widowed | 0.68 |
|  | (0.22 - 2.09) |
| Single or divorced | 0.86 |
|  | (0.58 - 1.27) |
| Migration background (ref: no) | 1.52 |
|  | (0.94 - 2.47) |
| Having children (ref: no) | 0.93 |
|  | (0.65 - 1.32) |
| Having grandchildren (ref: no) | 1.07 |
|  | (0.65 - 1.76) |
| ***Access factors*** |  |
| Private health insurance (ref: statutory health insurance) | 0.45* |
|  | (0.22 - 0.90) |
| *Internet connection quality at home (ref: fast and stable)* |  |
| Fast, but not stable | 0.92 |
|  | (0.57 - 1.50) |
| Stable, but not fast | 1.24 |
|  | (0.76 - 2.01) |
| Neither fast nor stable or no internet connection at home | 1.04 |
|  | (0.55 - 1.95) |
| ***Health factors*** |  |
| Depressive symptoms | 1.03 |
|  | (0.98 - 1.07) |
| Anxiety symptoms | 1.00 |
|  | (0.95 - 1.06) |
| Presence of at least one chronic physical illness (ref: no) | 1.02 |
|  | (0.73 - 1.42) |
| Self-rated health | 1.20 |
|  | (0.95 - 1.53) |
| ***COVID-19-related factors*** |  |
| Received COVID-19 vaccination (ref: no) | 0.80 |
|  | (0.50 - 1.27) |
| Fear of COVID-19 | 0.99 |
|  | (0.96 - 1.01) |
| ***Psychosocial factors*** |  |
| Loneliness | 1.44* |
|  | (1.06 - 1.96) |
| Perceived social support by family and friends | 1.04* |
|  | (1.00 - 1.07) |
| Life satisfaction | 1.02 |
|  | (0.99 - 1.06) |
| Self-efficacy | 0.71** |
|  | (0.56 - 0.89) |
| Attitude toward telemental health services | 1.03*** |
|  | (1.01 - 1.05) |
| ***Personality*** |  |
| Conscientiousness | 0.96 |
|  | (0.91 - 1.01) |
| Extraversion | 1.02 |
|  | (0.98 - 1.07) |
| Agreeableness | 1.02 |
|  | (0.96 - 1.07) |
| Openness | 1.04 |
|  | (0.99 - 1.08) |
| Neuroticism | 0.96 |
|  | (0.90 - 1.02) |
| ***Provider characteristics*** |  |
| Provider attitude toward telemental health services | 1.92*** |
|  | (1.58 - 2.32) |
| Provider skills for using telemental health services | 1.50*** |
|  | (1.23 - 1.84) |
| Constant | 0.01*** |
|  | (0.00 - 0.13) |
|  |  |
| Observations | 983 |
| Pseudo R-squared | 0.253 |

*Note.* Odds Ratios are reported with 95% confidence interval in parentheses. *** p<0.001, ** p<0.01, * p<0.05.

Table S5. Results of logistic regression for determinants of telemental health service use among patients with an affective disorder diagnosis since the COVID-19 pandemic (n=1,470).

| **Variables** | **Values** |
| --- | --- |
|  |  |
| ***Socioeconomic factors*** |  |
| *Gender (ref: men)* |  |
| Women | 0.92 |
|  | (0.69 - 1.22) |
| Diverse or intersex | 0.14 |
|  | (0.01 - 2.29) |
| Age | 0.98*** |
|  | (0.96 - 0.99) |
| *Educational level (ref: low educational level)* |  |
| Medium educational level | 0.97 |
|  | (0.64 - 1.47) |
| High educational level | 1.13 |
|  | (0.73 - 1.75) |
| *Employment status (ref: unemployed)* |  |
| Full-time employed | 1.04 |
|  | (0.74 - 1.45) |
| Part-time employed | 1.40 |
|  | (0.98 - 2.00) |
| Other | 1.02 |
|  | (0.62 - 1.69) |
| *Household income (ref: low income)* |  |
| Medium income | 1.00 |
|  | (0.72 - 1.37) |
| High income | 1.03 |
|  | (0.69 - 1.55) |
| *Area lived in (ref: urban)* |  |
| Mostly urban | 0.85 |
|  | (0.65 - 1.12) |
| Rural | 0.93 |
|  | (0.64 - 1.36) |
| *Living situation (ref: living with partner in the same household)* |  |
| Living with partner without a common household | 0.68 |
|  | (0.39 - 1.20) |
| Partner deceased or widowed | 1.01 |
|  | (0.46 - 2.20) |
| Single or divorced | 0.92 |
|  | (0.67 - 1.26) |
| Migration background (ref: no) | 1.18 |
|  | (0.78 - 1.78) |
| Having children (ref: no) | 1.12 |
|  | (0.85 - 1.49) |
| Having grandchildren (ref: no) | 0.92 |
|  | (0.62 - 1.37) |
| ***Access factors*** |  |
| Private health insurance (ref: statutory health insurance) | 0.87 |
|  | (0.51 - 1.47) |
| *Internet connection quality at home (ref: fast and stable)* |  |
| Fast, but not stable | 0.92 |
|  | (0.64 - 1.34) |
| Stable, but not fast | 1.24 |
|  | (0.82 - 1.87) |
| Neither fast nor stable or no internet connection at home | 1.19 |
|  | (0.75 - 1.90) |
| ***Health factors*** |  |
| Depressive symptoms | 1.03 |
|  | (1.00 - 1.07) |
| Anxiety symptoms | 1.01 |
|  | (0.97 - 1.06) |
| Presence of at least one chronic physical illness (ref: no) | 1.02 |
|  | (0.78 - 1.33) |
| Self-rated health | 1.04 |
|  | (0.86 - 1.26) |
| ***COVID-19-related factors*** |  |
| Received COVID-19 vaccination (ref: no) | 0.81 |
|  | (0.55 - 1.20) |
| Fear of COVID-19 | 1.00 |
|  | (0.97 - 1.02) |
| ***Psychosocial factors*** |  |
| Loneliness | 1.24 |
|  | (0.98 - 1.58) |
| Perceived social support by family and friends | 1.02 |
|  | (1.00 - 1.05) |
| Life satisfaction | 1.01 |
|  | (0.99 - 1.04) |
| Self-efficacy | 0.93 |
|  | (0.77 - 1.12) |
| Attitude toward telemental health services | 1.03*** |
|  | (1.02 - 1.04) |
| ***Personality*** |  |
| Conscientiousness | 1.01 |
|  | (0.97 - 1.06) |
| Extraversion | 1.00 |
|  | (0.97 - 1.04) |
| Agreeableness | 1.01 |
|  | (0.97 - 1.05) |
| Openness | 1.01 |
|  | (0.98 - 1.05) |
| Neuroticism | 0.98 |
|  | (0.93 - 1.03) |
| ***Provider characteristics*** |  |
| Provider attitude toward telemental health services | 2.00*** |
|  | (1.71 - 2.33) |
| Provider skills for using telemental health services | 1.36*** |
|  | (1.16 - 1.60) |
| Constant | 0.01*** |
|  | (0.00 - 0.04) |
|  |  |
| Observations | 1,470 |
| Pseudo R-squared | 0.219 |

*Note.* Odds Ratios are reported with 95% confidence interval in parentheses. *** p<0.001, ** p<0.01, * p<0.05.
